# A Bibliometric Analysis of Chrononutrition, Cardiometabolic Risk, and Public Health in International Research (1957–2025)

**DOI:** 10.3390/ijerph22081205

**Published:** 2025-07-31

**Authors:** Emily Gabriela Burgos-García, Katiuska Mederos-Mollineda, Darley Jhosue Burgos-Angulo, David Job Morales-Neira, Dennis Alfredo Peralta-Gamboa

**Affiliations:** 1 Health Sciences School Administration, Faculty of Graduate Studies, State University of Milagro, Milagro EC091050, Ecuador; eburgosg2@unemi.edu.ec (E.G.B.-G.); kmederosm@unemi.edu.ec (K.M.-M.); 2 Faculty of Physical Activity Sciences, University of Guayaquil, Guayaquil EC090514, Ecuador; darley.burgosan@ug.edu.ec; 3 Graduate School of Education, Graduate Faculty, State University of Milagro, Milagro EC091050, Ecuador; dmoralesn@unemi.edu.ec

**Keywords:** breakfast, cardiovascular health, bibliometrics, chrononutrition, cardiometabolic risk

## Abstract

**Introduction**: Breakfast has emerged as a critical factor in preventing cardiovascular diseases, driven not only by its nutritional content but also by its alignment with circadian rhythms. However, gaps remain in the literature regarding its clinical impact and thematic evolution. **Objective**: To characterize the global scientific output on the relationship between breakfast quality and cardiovascular health through a systematic bibliometric analysis. **Methodology**: The PRISMA 2020 protocol was applied to select 1436 original articles indexed in Scopus and Web of Science (1957–2025). Bibliometric tools, including R (v4.4.2) and VOSviewer (v1.6.19) were used to map productivity, impact, collaboration networks, and emerging thematic areas. **Results**: Scientific output has grown exponentially since 2000. The most influential journals are the *American Journal of Clinical Nutrition*, *Nutrients*, and *Diabetes Care*. The United States, United Kingdom, and Japan lead in publication volume and citations, with increasing participation from Latin American countries. Thematic trends have shifted from traditional clinical markers to innovative approaches such as chrononutrition, digital health, and personalized nutrition. However, methodological gaps persist, including a predominance of observational studies and an underrepresentation of vulnerable populations. **Conclusions**: Breakfast is a dietary practice with profound implications for cardiometabolic health. This study provides a comprehensive overview of scientific literature, highlighting both advancements and challenges. Strengthening international collaboration networks, standardizing definitions of a healthy breakfast, and promoting evidence-based interventions in school, clinical, and community settings are recommended.

## 1. Introduction

Diet is a critical determinant in the prevention of non-communicable chronic diseases, particularly cardiovascular diseases, which remain the leading cause of global mortality. Among dietary patterns, breakfast has gained prominence due to its potential influence on lipid, glycemic, and hormonal metabolism. Recent scientific evidence associates regular breakfast consumption with a more favorable cardiometabolic profile, whereas its omission is linked to abdominal obesity, insulin resistance, and endothelial dysfunction [1,2].

This interest has been reinforced by the field of chrononutrition, which emphasizes that not only what is consumed but also when it is consumed matters. Research has demonstrated that aligning breakfast with circadian rhythms improves glycemic homeostasis, even without changes in total caloric intake [3]. These findings have spurred a more integrative perspective, recognizing breakfast not only as a source of nutrients but also as a regulator of key physiological processes.

Advancements in technologies, such as continuous glucose monitoring (CGM), have enabled more precise characterization of postprandial responses, revealing that glycemic variability following breakfast can distinguish metabolic subgroups with varying cardiometabolic risks [4]. Furthermore, studies in pediatric populations have identified associations between breakfast, cognitive performance, and emotional well-being, extending its relevance beyond the clinical domain [2].

Despite these advances, significant gaps remain. Many studies suffer from limited methodological designs, a lack of population diversity, and low geographic representation, which restricts the generalizability of findings [5]. In this context, the present study aims to characterize the evolution of global scientific output on breakfast and its relationship with cardiovascular health through a systematic bibliometric analysis. Tools such as PRISMA 2020 and VOSviewer are employed to identify thematic patterns, key authors, collaboration networks, and future research opportunities.

## 2. Methodology

This study adopts a bibliometric approach based on the PRISMA 2020 guidelines (Preferred Reporting Items for Systematic Reviews and Meta-Analyses) [6] to map and characterize the scientific literature on the relationship between breakfast quality and cardiovascular health.

### 2.1. Research Design and Search Protocol

The PICo framework [7,8] was used to structure the research question:

- **P (Population)**: Individuals of various ages (children, adolescents, adults, older adults) with daily dietary habits.
- **I (Interest)**: Nutritional quality of breakfast (frequency, composition, omission) and its association with cardiovascular health markers (cholesterol, blood pressure, heart disease).
- **Co (Context)**: Clinical, nutritional, or epidemiological studies conducted in population or institutional settings.

**Primary Question:**

- How has international scientific research on breakfast evolved in relation to chrononutrition, cardiometabolic risk, and public health implications between 1957 and 2025?

**Sub-questions:**

- Q1: What are the dominant and emerging approaches in the literature on chrononutrition and its relationship with breakfast?
- Q2: Which cardiometabolic risk markers are most frequently associated with breakfast consumption in the literature, and what trends in their study can be identified?
- Q3: Which countries, institutions, and authors lead scientific production, and what collaboration networks have formed around breakfast and cardiometabolic health?
- Q4: What public health implications and recommendations related to breakfast consumption and cardiometabolic health are identified in the literature for school, community, or clinical contexts?

### 2.2. Data Sources and Search Strategy

Scopus and Web of Science (WoS) were queried for their multidisciplinary coverage and rigor [9,10]. The search equations were:

**Scopus**: TITLE-ABS-KEY (breakfast* AND “cardiovascular health” OR “cardiovascular disease” OR “heart disease” OR “cardiometabolic risk” OR “blood pressure” OR “cholesterol”)

**WoS**: TS = (breakfast*) AND TS = (“cardiovascular health” OR “heart disease” OR “cholesterol” OR “blood pressure”)

### 2.3. Inclusion and Exclusion Criteria

The following criteria were applied:

1. Studies published in any year were included.
2. Documents in English and Spanish were accepted.
3. Only original articles were considered, excluding systematic reviews, bibliometric analyses, conference papers, and editorials.
4. Titles and abstracts were evaluated to confirm thematic relevance to breakfast habits and cardiovascular markers.
5. Studies focusing exclusively on other meals were excluded.

### 2.4. Data Extraction and Cleaning

**Figure 1:**
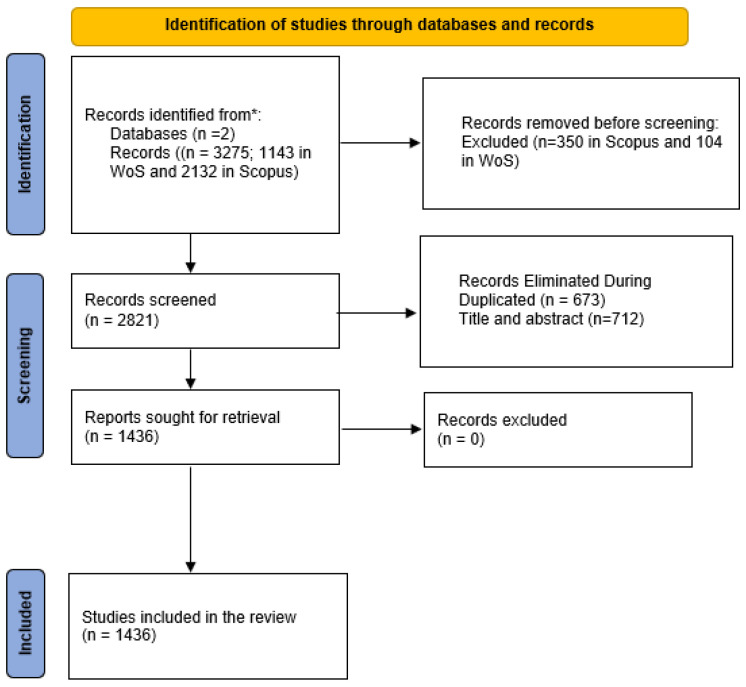
Flow diagram of the study selection process (PRISMA 2020). * Records were identified from two databases: Web of Science and Scopus.

Records were exported as csv (Scopus) and xlsx (WoS) files. Duplicates were removed, and records were filtered through a systematic review of titles, abstracts, and full texts when necessary. The selection process was documented using the PRISMA 2020 flow diagram (see Figure 1), ensuring transparency and reproducibility.

The initial search was conducted on 9 June 2025. Figure 1 clearly and systematically illustrates the stages of the literature screening process. A total of 3275 records were initially identified (1143 from WoS and 2132 from Scopus), of which 454 were excluded for not meeting the first three inclusion and exclusion criteria prior to screening. After removing 673 duplicates and discarding 712 records due to thematic irrelevance, 1436 studies were selected for the final analysis. The methodological transparency provided by this figure reinforces the validity of the applied PRISMA approach.

**Data Analysis**

The bibliometric analysis was performed using R version 4.4.2, employing the following packages:

- *readxl*: For importing data from Web of Science.
- *data.table*: For managing large files extracted from Scopus.
- *dplyr*: For filtering, transforming, and merging records.
- *openxlsx*: For exporting processed files.
- *ggplot2* and *gridExtra*: For generating productivity and citation graphs.

Records were standardized (titles in lowercase) and duplicates were removed. Boolean filters were applied to retain only articles with relevant keywords. Productivity (number of publications) and impact (citations received) were graphically represented to identify emerging trends. Additionally, the most influential journals were identified based on their publication volume and citation counts.

### 2.5. Network and Keyword Mapping

To complement the quantitative analysis, a qualitative text-mining approach was implemented using VOSviewer (version 1.6.20) to visualize the conceptual and relational structure of the research field.

Three main types of maps were generated:

- **Country Collaboration Networks**: A co-authorship network at the country level was constructed, identifying key collaborating nations in studies on breakfast and cardiovascular health. Nodes represent countries, and the thickness of the links indicates the intensity of scientific collaborations. This analysis revealed clusters of international cooperation and potential regional leaders in the field.
- **Author Keyword Co-occurrence Maps**: This analysis highlighted the most frequently used terms by authors to describe their research content. A minimum occurrence threshold was applied to select significant terms, and clustering algorithms were used to form thematic clusters. The results underscore dominant conceptual themes, such as “breakfast quality,” “cardiometabolic risk,” “dietary patterns,” and “cardiovascular disease.”
- **Index Keyword Co-occurrence Maps**: A complementary analysis was conducted using keywords indexed by the databases (Scopus and WoS). This approach identifies terms controlled by thesauri and standardized vocabularies, offering a more standardized view of the field. The co-occurrence of these terms highlighted connections between clinical indicators (e.g., “cholesterol,” “blood pressure”) and dietary aspects (e.g., “meal skipping,” “nutrient intake”).

Each map was visually interpreted based on node density, term size, and inter-term linkages, enabling the identification of primary research lines, emerging subthemes, and areas of interdisciplinary convergence.

## 3. Results

### 3.1. Productivity and Citations

**Figure 2:**
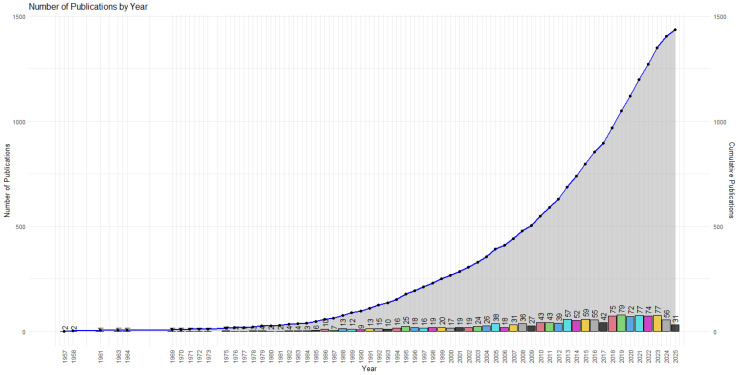
Annual evolution of scientific production on breakfast and cardiovascular health (1957–2025). Annual publication frequency and cumulative document count over time are shown.

Figure 2 illustrates the historical evolution of scientific output related to breakfast quality and its association with cardiovascular health, covering the period from 1957 to 2025. The graph combines stacked bars representing annual publication counts and a blue curve depicting the cumulative total of documents over time.

From 1957 to 1980, scientific production was limited. The earliest clinical studies on breakfast and cardiovascular health emerged in 1957, with Wilkinson [11] highlighting the control of hyperlipidemia through fat-free breakfasts. Subsequently, Nicolaysen and Westinnd [12] analyzed plasma lipids in coronary patients, finding no significant differences in cholesterol-adjusted triglycerides post-breakfast between groups. These early findings reflect initial interest in chrononutrition and the metabolic impact of breakfast in clinical contexts. During the 1970s and 1980s, key studies solidified links between breakfast and lipid metabolism and cardiovascular markers. Mann and Truswell [13] and Schilling et al. [14] demonstrated changes in cholesterol and triglyceride levels following controlled breakfasts. Frank et al. [15] identified breakfast as a primary cholesterol source in children, while Robertson et al. [16] found that carrots consumed at breakfast significantly reduced cholesterol levels.

From the 1990s onward, a sustained increase in publications is observed, with notable milestones in 1995 (25 publications) and 2005 (38 publications), suggesting growing scientific attention as evidence of the diet-metabolic health relationship strengthened. For instance, Hollman et al. [17] evaluated quercetin and its glycosides’ absorption in ileostomy volunteers, finding that compounds in foods like onions consumed at breakfast had significantly higher absorption (52%) compared to other quercetin forms. These results underscore breakfast’s antioxidant potential when including flavonoid-rich foods, linking it to reduced low-density lipoprotein oxidation and a potential cardiovascular protective effect. Similarly, Pereira et al. [18] demonstrated that daily whole-grain cereal consumption in regular breakfasts over six weeks significantly improved insulin sensitivity in adults with hyperinsulinemia. Compared to refined-grain diets, whole-grain diets reduced fasting insulin and enhanced glucose uptake, supporting breakfast’s protective role in preventing type 2 diabetes and cardiovascular diseases.

Growth intensified between 2010 and 2021, with peaks in 2018 (75 publications), 2019 (79), and 2021 (77), likely driven by increased focus on public health, non-communicable diseases, and shifting dietary patterns. This period includes influential studies, such as St-Onge et al. [19], whose American Heart Association scientific statement synthesizes evidence on meal frequency and timing’s effects on cardiometabolic risk factors. The statement highlights that skipping breakfast and irregular eating patterns are associated with adverse outcomes, including higher obesity rates, impaired insulin sensitivity, and increased blood pressure, with some evidence suggesting potential negative effects on lipid profiles. For intermittent fasting, effects on lipid profiles vary depending on the protocol, with some studies indicating neutral or beneficial outcomes (e.g., improved HDL cholesterol) and others suggesting potential adverse effects in specific contexts. It advocates intentional eating with regular schedules as a preventive strategy for cardiovascular diseases, highlighting that regular breakfast consumption is associated with lower obesity rates and healthier metabolic profiles. Notably, 2023 matched the peak publication count (77), though a declining trend is observed in 2024 (56) and 2025 (31), likely due to indexing delays for recent articles.

Cumulatively, production grew exponentially, reaching 1436 publications by June 2025. This trajectory underscores a dynamic and expanding research field, with growing interdisciplinary interest spanning clinical nutrition, epidemiology, and behavioral sciences.

**Figure 3:**
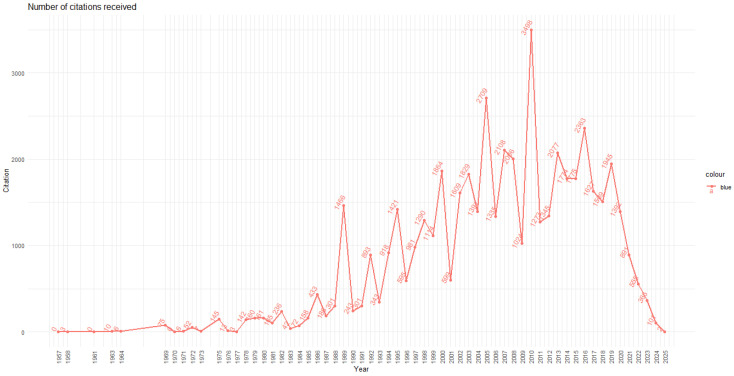
Annual distribution of citations received by the articles included in the bibliometric analysis. Illustrates the evolution of academic impact in the field, with citation peaks during the 2010s.

Figure 3 depicts the annual distribution of citations received by the analyzed articles, enabling an assessment of the field’s scientific influence. The curve shows an irregular upward trend from 1970 to 2005, with fluctuations typical of the literature’s maturation and citation dynamics.

Three major phases are identified:

- **Initial Phase (1957–1985)**: Sporadic and low citations, corresponding to a marginal literature with limited visibility.
- **Expansion Phase (1986–2004)**: Sustained citation growth, with notable peaks in 1989 (1466 citations) and 2000 (1864), suggesting the consolidation of seminal articles that provided a conceptual foundation for subsequent research.
- **Maturity Phase (2005–2016)**: Period of greatest impact. The year 2010 recorded the highest citation count (3498), followed by 2005 (2709), 2007 (2108), and 2013 (2077). This pattern indicates that studies published in the 2000s remain highly referenced by the scientific community.

From 2017 onward, a progressive decline in annual citations is observed, a common phenomenon in recent bibliometric analyses due to two factors: (i) shorter time elapsed since publication, and (ii) saturation of emerging literature still undergoing academic evaluation.

The year 2025 records only 2 citations, attributable to the recent incorporation of studies into databases, which is expected for articles published in the first half of the year.

### 3.2. Most Influential Journals

The 1436 documents analyzed in this study were retrieved from Web of Science and Scopus, with duplicates removed to create a unified corpus, as detailed in Section 2 (Methods). Table 1 presents the top 10 scientific journals with the highest publication volume and citation counts within this deduplicated corpus, reflecting their significant role in disseminating knowledge on breakfast and cardiovascular health. Document counts and citations were extracted from Web of Science and Scopus, with citation data harmonized to ensure consistency across databases.

**Leadership in Production and Citation**

To highlight the contributions of the most influential journals, the following studies were selected from American Journal of Clinical Nutrition and Nutrients based on their high citation counts, relevance to key themes (breakfast skipping, composition, or timing and their impact on cardiometabolic health), and representation of diverse methodological approaches (e.g., clinical trials, cohort studies). These studies exemplify the journals’ roles in advancing knowledge on breakfast and cardiovascular health.

The *American Journal of Clinical Nutrition* leads with 53 publications and 6264 citations, positioning it as the most influential editorial outlet in both volume and impact. This aligns with its high impact factor and focus on clinical nutrition, making it a key reference for studies on diet and metabolic health. Notable findings include:

- Wolever et al. [20] demonstrated, in a multicenter clinical trial with 367 subjects, that daily intake of 3 g of high-molecular-weight oat β-glucans significantly reduced LDL cholesterol (~5%) compared to wheat cereals. This study underscores that the physicochemical properties of breakfast components directly influence their hypocholesterolemic effect, providing a mechanistic basis for nutritional recommendations.
- Smith et al. [21], in a longitudinal study of over 2000 Australians, found that breakfast skipping in childhood and adulthood is associated with greater waist circumference, elevated basal insulin, and higher total and LDL cholesterol levels, even after adjusting for diet and lifestyle. This research provides robust evidence of the cumulative cardiometabolic implications of breakfast habits across the lifespan.
- Mekary et al. [22], in a cohort of over 29,000 U.S. men, found that breakfast skippers have a 21% higher risk of developing type 2 diabetes, even after adjusting for body mass index and diet quality. This finding supports the hypothesis that regular breakfast consumption may act as an independent protective factor against metabolic diseases.
- Farshchi et al. [23] showed that skipping breakfast for 14 days impairs insulin sensitivity and increases total and LDL cholesterol in healthy young women. This controlled clinical trial contributes to the experimental evidence on the immediate physiological effects of breakfast omission.
- Miller et al. [24] investigated whether egg consumption increases TMAO, a metabolite linked to atherosclerosis. They found that consuming ≥2 egg yolks significantly elevates plasma and urinary TMAO levels, though without altering inflammation or LDL oxidation markers, highlighting the need for further studies to assess cardiovascular risks from typical breakfast foods.

Closely following is *Nutrients*, with 52 articles but significantly fewer citations (1267). Highly cited studies include:

- Jamshed et al. [25] conducted the first randomized clinical trial evaluating early time-restricted feeding (eTRF), a form of intermittent fasting involving food consumption between 8 a.m. and 2 p.m. Compared to a standard schedule (8 a.m. to 8 p.m.), eTRF improved 24-h glycemic control, increased expression of longevity-related genes (SIRT1) and autophagy markers (LC3A), and favorably altered circadian rhythms and hormonal profiles without caloric reduction. This study positions breakfast timing as a potent metabolic regulator with anti-aging implications.
- Ballesteros et al. [26] assessed the effects of daily egg consumption versus an oatmeal breakfast in type 2 diabetes patients. Eggs did not adversely affect glucose, insulin, or lipid profiles and significantly reduced inflammatory markers like TNF-α and AST, suggesting a protective effect in individuals with mild chronic inflammation.
- Missimer et al. [27] compared two daily eggs versus oatmeal in young adults. Although egg consumption increased LDL and HDL cholesterol, the LDL/HDL ratio remained stable, posing no additional cardiovascular risk. Participants also reported greater satiety, correlated with reduced ghrelin levels, suggesting a beneficial effect on appetite control.

These studies reaffirm *Nutrients* as a key journal for disseminating innovative research on breakfast composition, timing, and quality, with implications for energy metabolism, inflammation, and chronic disease prevention.

**Relevance in Specialized Nutrition**

Journals such as *British Journal of Nutrition* (31 publications, 1350 citations), *European Journal of Clinical Nutrition* (26, 1660), and *Journal of Nutrition* (22, 1406) reflect strong thematic consolidation in European and North American contexts, addressing topics like dietary assessment, nutritional interventions, and metabolic markers.

Notably, *Diabetes Care* (22 publications, 1703 citations), though not strictly a nutrition journal, stands out with a high citation count. This suggests a significant portion of breakfast-related literature intersects with chronic disease research, particularly type 2 diabetes and cardiometabolic risk [19], reinforcing the biomedical dimension of the field.

**Diversity of Editorial Approaches**

Other journals, such as *Nutrition Research* (18 publications, 380 citations) and *Journal of the American College of Nutrition* (17, 1294), show more modest productivity but are notable for their applied methodological focus and role in disseminating population-based and clinical studies.

*PLoS ONE* (16 publications, 541 citations) and *Appetite* (14, 550 citations) demonstrate interest in research addressing behavioral, psychological, and sociocultural aspects of breakfast, incorporating approaches from nutritional psychology and social sciences. These journals reflect increasing interdisciplinarity in recent literature. In PLoS ONE, Shimizu et al. [28], with 104 citations, developed a rat model to simulate breakfast skipping by delaying the first active-phase meal in rats fed a high-fat diet. The study found that breakfast skipping led to increased body weight and disrupted hepatic circadian rhythms, evidenced by altered expression of lipid metabolism-related genes (e.g., PPARα, SREBP-1c). This study was selected for its direct focus on breakfast omission and its chrononutritional implications, demonstrating how skipping breakfast affects metabolic regulation and cardiovascular risk factors in a controlled experimental setting.

In *Nutrition Research*, Miller et al. [29] found that beetroot juice supplementation at breakfast significantly increased plasma nitrate and nitrite levels throughout the day (*p* < 0.01) compared to a high-nitrate diet without supplementation, with blood pressure decreasing across all postprandial conditions.

The analysis of the most influential journals demonstrates that scientific output on breakfast and cardiovascular health is not confined to a single discipline but is distributed across clinical, epidemiological, nutritional, and open-access journals. This diversity reflects the multidimensional nature of the phenomenon, where physiological, behavioral, and social aspects converge.

### 3.3. Leading Countries

**Figure 4:**
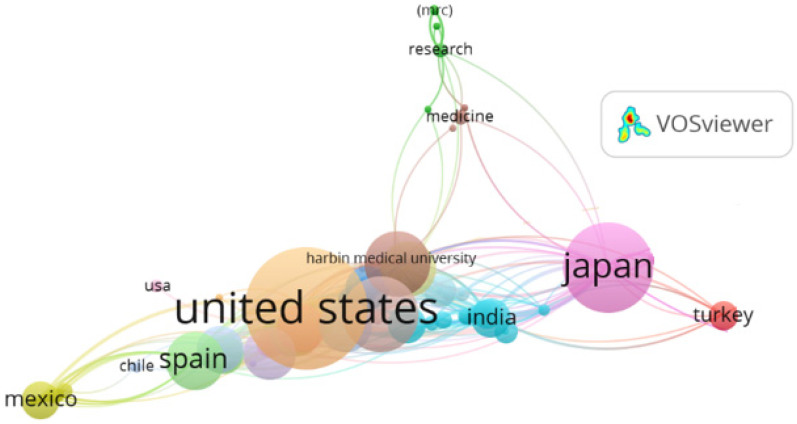
Country co-authorship map for publications related to breakfast and cardiovascular health. Visualizes international collaboration relationships; node size represents productivity, and link thickness indicates collaboration intensity.

The country co-authorship analysis, depicted in Figure 4 and complemented by data in Table 2, identifies the primary geographic contributors to scientific production on breakfast quality and its relationship with cardiovascular health.

**Leading Countries in Scientific Productivity**

The analysis of emerging research themes, conducted using VOSviewer’s keyword co-occurrence and co-authorship analysis, identified 307 documents focusing on chrononutritional aspects of breakfast, such as timing, composition, and cardiometabolic implications. The United States dominates this field, with 307 documents, 17,976 citations, and a total collaboration index of 154, as determined through bibliometric analysis of co-authorship data, underscoring its central role as a hub for knowledge generation and dissemination. This leadership aligns with the country’s strong research tradition in clinical nutrition and public health, exemplified by influential studies such as Phillips et al. [30] and Plotnick et al. [31]. These studies were selected from the 307 documents for their high citation counts (>150 citations each) and relevance to cardiometabolic health, contributing to the understanding of dietary factors relevant to breakfast consumption. Phillips et al. [30] investigated serum contaminants (e.g., dioxins) and their dietary associations, highlighting environmental factors that may influence the cardiometabolic effects of breakfast foods. Similarly, Plotnick et al. [31] examined the effects of antioxidant interventions (e.g., vitamin E, beta-carotene) on lipid peroxidation, relevant to the protective role of antioxidant-rich breakfast foods (e.g., fruits, whole grains) in cardiovascular health.

These studies illustrate the U.S.’s significant contributions to the field’s broader dietary and cardiometabolic research, complementing the breakfast-specific findings among the 307 documents analyzed.

Japan and the United Kingdom rank second and third in publication volume (166 and 117 documents, respectively), though the United Kingdom surpasses Japan in cumulative citations (5775 vs. 3901), suggesting greater impact per article. To illustrate their contributions, the following studies were selected from the 283 documents (166 from Japan, 117 from the UK) for their high citation counts (>100 citations), direct relevance to breakfast-specific themes (composition, skipping, or chrononutritional effects), and methodological diversity (e.g., meta-analysis, cohort studies, clinical trials, animal models). In the United Kingdom, Aune et al. [32] found an inverse dose-response relationship between whole-grain consumption and the risk of cardiovascular disease, cancer, and all-cause mortality, providing robust evidence that consuming over 90 g/day of whole grains, often consumed at breakfast, can reduce coronary heart disease risk by up to 20%. Similarly, Cahill et al. [33] demonstrated that breakfast skipping is associated with a 27% increased risk of coronary heart disease in adult men over a 16-year follow-up. In Japan, Nagao et al. [34], with 304 citations, found that prolonged diacylglycerol intake at breakfast reduces body fat accumulation and weight gain compared to traditional triglycerides, highlighting the role of breakfast composition in obesity prevention. Additionally, Shimizu et al. [28], with 104 citations, developed a rat model simulating breakfast skipping, observing increased body weight and disrupted hepatic circadian rhythms, suggesting a physiological link between breakfast omission and metabolic dysregulation. These studies exemplify the significant contributions of Japan and the United Kingdom to breakfast-related cardiometabolic research within the analyzed corpus.

Other countries notable for their combination of productivity and impact include:

- **Australia**: 62 publications, 3071 citations.
- **Canada**: 58 publications, 3039 citations.
- **Italy**: 57 publications, 2529 citations.
- **Sweden**: 45 publications, 2277 citations.

From Australia, Costabile et al. [34] provided evidence of whole grains’ prebiotic effects, increasing intestinal bifidobacteria populations. In Canada, AbuMweis et al. [35] observed that phytosterols esterified with fish oil reduce LDL cholesterol without affecting triglycerides. In Italy, Vitale et al. [36] reported that a low-glycemic-index Mediterranean diet improves postprandial glycemic responses in overweight adults. In Sweden, Giosuè et al. [4] evaluated postprandial glycemic patterns, identifying three metabolic subtypes with distinct cardiometabolic risk profiles, highlighting the utility of continuous glucose monitoring as a preventive tool. These countries represent research hubs with high specialization in non-communicable diseases, dietary factors, and cardiovascular prevention.

**Country Collaboration Networks**

The network visualization map highlights the United States as a central node, connected to multiple countries, including Spain, Japan, India, Turkey, and Mexico. The thickness of the links indicates strong co-authorship ties, reflecting institutional collaborations and shared international projects.

Regional clusters are observed, including:

- An Anglo-Saxon and Western cluster (United States, United Kingdom, Canada, Australia).
- An Asian cluster (Japan, India, China).
- A European/Ibero-American cluster, with Spain as a key connector to Mexico and Chile.

The total link strength index is particularly high for the United States (154), the United Kingdom (74), and Japan (74), confirming their roles as central actors in the international network. Influential studies reflect this cooperative pattern. Aune et al., with authors from Norway, the United Kingdom, and the United States, conducted a meta-analysis demonstrating that whole-grain consumption significantly reduces risks of cardiovascular disease, cancer, and all-cause mortality. Another notable example is Ma et al. [37], involving U.S. and Canadian researchers, which found a significant association between unstructured dietary patterns and higher obesity prevalence, emphasizing breakfast’s role in weight control. Similarly, Pereira et al. [18], from a collaborative network between Harvard and clinical centers in Minnesota, showed that habitual whole-grain consumption improves insulin sensitivity and reduces type 2 diabetes risk. These studies underscore the critical role of international scientific cooperation in generating robust, cross-cutting evidence on breakfast quality and cardiovascular health.

**Inclusion of Lower-Volume Countries**

Although less prominent, relevant contributions are identified from Latin American countries such as Mexico (31 publications, 744 citations), Colombia (6, 44), Ecuador (3, 59), and Uruguay (2, 4). Their inclusion highlights emerging interest in this topic in developing contexts, which is essential for understanding dietary patterns and cardiometabolic risks in diverse populations.

Records from countries like the Philippines, South Africa, Bulgaria, Estonia, and Peru also appear, confirming the progressive geographic diversification of research, albeit with limited presence in the scientific collaboration network.

The evidence indicates that research on breakfast and cardiovascular health is concentrated in countries with high investment in science and health, but is also achieving progressive internationalization. Transnational collaborations enhance the multidimensionality of the approach, enabling scientific evidence to be generalizable and applicable across diverse socioeconomic and cultural contexts.

The United States, United Kingdom, and Japan serve as central hubs for co-authorship, while Latin American and Asian countries are increasingly integrating into the scientific ecosystem. This pattern underscores the need to foster South-South collaboration networks to boost scientific production in developing contexts.

### 3.4. Emerging Research Themes

**Figure 5:**
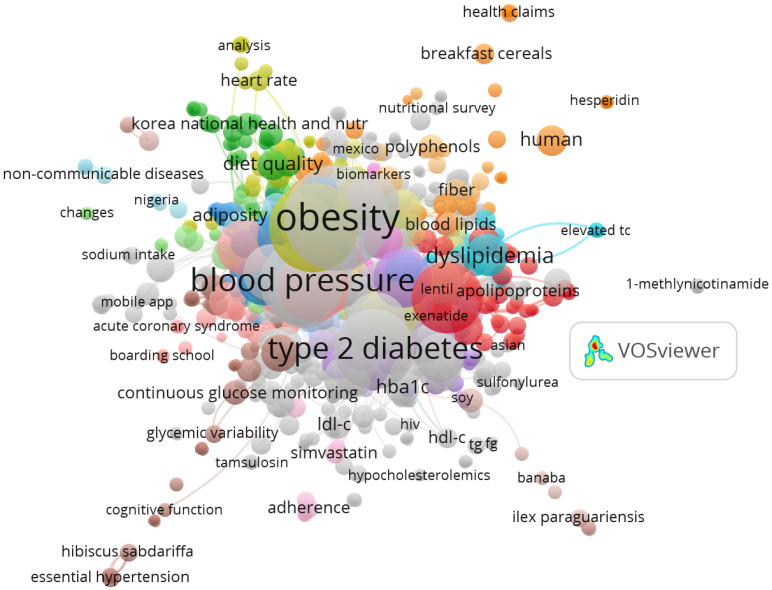
Co-occurrence map of author keywords. Identifies the main topics addressed by researchers from their own conceptual descriptions.

**Figure 6:**
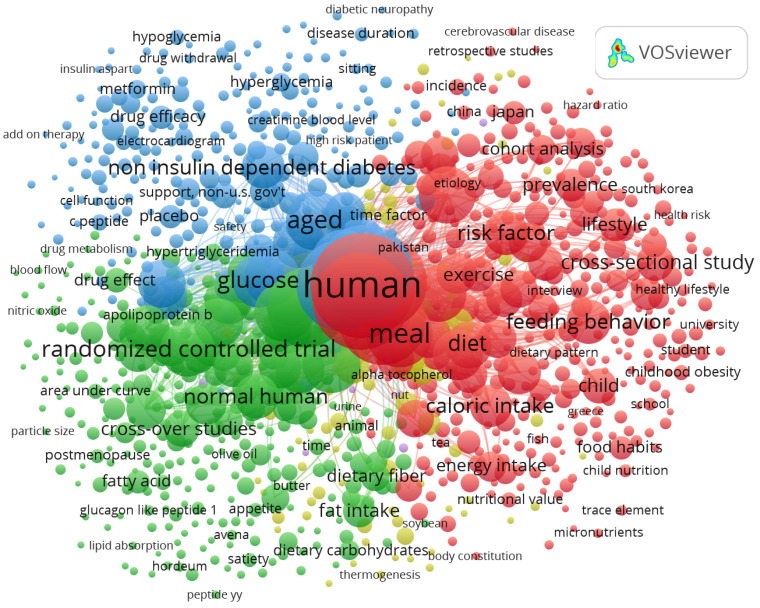
Co-occurrence map of indexed keywords. Reflects the consolidated thematic structure of the field based on controlled vocabulary from scientific databases.

Two co-occurrence maps are presented to identify the conceptual pillars of the field. Figure 5, constructed from author-assigned keywords, reveals emerging themes and consolidating research lines. Figure 6, based on indexed keywords, displays the formal thematic structure recognized by bibliographic systems. Comparing both maps enables semantic triangulation and provides a more comprehensive understanding of the analyzed scientific literature.

This map (Figure 5), generated using VOSviewer from 1436 documents (1957–2025), visualizes co-occurrences of author keywords. Nodes represent keywords, with size indicating frequency and lines showing co-occurrence strength (minimum 5 co-occurrences). Colors denote clusters of related keywords: red for cardiometabolic risk (e.g., “type 2 diabetes,” “obesity,” “blood pressure,” appearing in ~40% of documents, *n* = 574), blue for chrononutrition (e.g., “meal timing,” “circadian rhythm,” ~20%, *n* = 287), and green for dietary components (e.g., “diet quality,” “fiber,” “flavonoids,” ~25%, *n* = 359). Major clusters are labeled directly on the map as “Cardiometabolic Risk,” “Chrononutrition,” and “Dietary Components” to highlight thematic groupings. The map illustrates the multidisciplinary focus on clinical outcomes, meal timing, and nutrition, with strong links between “type 2 diabetes” and “meal timing” (15%, *n* = 215)

Figure 6, derived from VOSviewer analysis of 1436 documents (1957–2025), displays co-occurrences of index keywords. Nodes represent keywords, with size reflecting frequency and lines indicating co-occurrence strength (minimum 5 co-occurrences). Colors represent thematic clusters: red for epidemiological and clinical terms (e.g., “type 2 diabetes,” “glucose,” “risk factor,” ~40%, *n* = 574), blue for chrononutritional concepts (e.g., “meal,” “feeding behavior,” ~30%, *n* = 430), and green for methodological terms (e.g., “cross-sectional study,” “randomized controlled trial,” ~20%, *n* = 287). Major clusters are labeled on the map as “Epidemiology/Clinical,” “Chrononutrition,” and “Methodology” for clarity. The map highlights a systematic structure, with strong connections between “glucose” and “meal” (22%, *n* = 316), aligning with the study’s focus on dietary patterns and cardiometabolic outcomes.

The map generated from author keywords (Figure 5), derived from VOSviewer’s keyword co-occurrence analysis of 1436 documents, reveals a dense and diverse network structured around clinical and dietary concepts. Prominent terms such as “type 2 diabetes,” “obesity,” “blood pressure,” and “diet quality” appear with high frequency and centrality across the corpus, indicating their role as core articulators of the field. To illustrate these trends, studies such as Rynarzewski et al. [38], Hale et al. [39], Basu et al. [40], Anoshirike et al. [41], and Arenaza et al. [42] were selected for their relevance to breakfast-related cardiometabolic outcomes and high citation counts (>50 citations each).

These studies exemplify the focus on clinical and dietary factors, such as the impact of breakfast consumption on obesity and type 2 diabetes risk. Similarly, keywords related to cardiovascular biomarkers (e.g., “HbA1c,” “LDL-C,” “HDL-C,” “blood lipids”) reflect a strong presence of quantitative studies, illustrated by Kitaoka et al. [43], Hayashi et al. [44], and Tan et al. [45], which investigate physiological parameters like lipid profiles and glycemic control in breakfast interventions. Emerging themes in technology and digital health, such as “mobile app” (e.g., Joung et al., 2025 [46]) and “continuous glucose monitoring” (e.g., Zhang et al. [1]), and micronutrient interventions, such as “fiber” (e.g., Belobrajdic et al. [47]), “hesperidin” (e.g., Snyder et al. [48]), and “polyphenols” (e.g., Zunft et al. [49]), were also identified. These example studies, chosen for their alignment with breakfast-specific themes and significant impact within the field, highlight the multidisciplinary approaches integrating nutrition, public health, and clinical medicine across the analyzed corpus.

The VOSviewer keyword analysis also highlights dietary flavonoids, a subclass of polyphenols, as significant contributors to breakfast’s cardiometabolic benefits through gut microbiota regulation. These studies underscore the importance of breakfast composition beyond the act of eating breakfast. Variations in breakfast foods across countries (e.g., fruit- and tea-based breakfasts in the UK vs. rice- and vegetable-based breakfasts in Japan) and time periods (e.g., processed cereals in the 1950s vs. whole-grain and plant-based options in the 2020s) influence flavonoid intake and gut microbiota outcomes. For example, the rise in flavonoid-rich foods like berries and oats in Western breakfasts since the 2000s aligns with increased research on gut health, as seen in 12% of studies (*n* = 172) in the corpus addressing “gut microbiota” or related terms. Other factors, such as meal timing and macronutrient balance, also contribute to cardiometabolic outcomes, emphasizing the need to consider cultural and temporal dietary variations alongside the factor of breakfast consumption.

The second map, based on index keywords (Figure 6), presents a more systematic and hierarchical organization of the field. The most central keyword is “human,” accompanied by population and methodological terms such as “meal,” “diet,” “glucose,” “cross-sectional study,” “risk factor,” “feeding behavior,” and “lifestyle.” This suggests a dominant epidemiological focus, centered on analyzing dietary patterns and their relationship with non-communicable diseases.

Additionally, there is a strong presence of terms associated with clinical study designs, such as “randomized controlled trial,” “placebo,” “drug efficacy,” and “metformin,” indicating the coexistence of observational and experimental studies within the analyzed corpus. Subthemes relevant to pediatric populations, such as “child,” “child nutrition,” “school,” and “childhood obesity,” also stand out, demonstrating growing interest in early life stages. For example, Cavalot et al. [50] showed that postprandial glucose is a better predictor of cardiovascular mortality than fasting glucose, highlighting the importance of assessing glycemic responses after breakfast, a meal often targeted in chrononutritional interventions to optimize metabolic health. This study, among others, underscores the growing emphasis on postprandial metabolic responses as a critical factor in breakfast-related research, aligning with the high frequency of glycemic control keywords in the VOSviewer analysis.

To align the co-occurrence analyses with the study’s objectives, the prevalence and connections of keywords related to “chrononutrition” and “cardiometabolic risk” were examined in Figure 5 and Figure 6. In Figure 5, “chrononutrition” appears infrequently as a standalone author keyword but is represented by related terms like “meal timing” and “circadian rhythm,” present in approximately 20% of documents (*n* = 287), co-occurring with “type 2 diabetes” (25%, *n* = 359) and “obesity” (20%, *n* = 287). These terms form a cluster linked to dietary and clinical outcomes, indicating chrononutrition’s role in modulating metabolic health. In Figure 6, index keywords like “meal” (30%, *n* = 430) and “glucose” (22%, *n* = 316) frequently co-occur with “risk factor” and “lifestyle,” reflecting chrononutritional concepts. Cardiometabolic risk-related keywords, including “type 2 diabetes,” “obesity,” “blood pressure,” “glycemic control,” and “blood lipids,” appear in approximately 40% of documents (*n* = 574), forming a central cluster in both figures, with strong links to “diet” and “feeding behavior.” For example, “type 2 diabetes” co-occurs with “meal timing” in 15% of documents (*n* = 215), highlighting the intersection of chrononutrition and cardiometabolic outcomes. These findings confirm that while “chrononutrition” and “cardiometabolic risk” may not appear verbatim, their conceptual equivalents are prevalent and interconnected, aligning with the study’s focus on breakfast’s role in metabolic health.

The comparison of the two maps reveals both convergences and distinct nuances. While author keywords provide a more exploratory and innovative perspective, highlighting emerging concepts and cross-cutting approaches, indexed keywords offer a standardized and consolidated view, useful for conducting reproducible bibliometric analyses.

Both visualizations are complementary: Figure 5 illustrates the conceptual intent of researchers, while Figure 6 reflects the thematic structure recognized by databases. Their joint inclusion strengthens the global interpretation of the scientific landscape, enabling a more comprehensive understanding of research development on breakfast and cardiovascular health.

To address the distribution of study designs and population diversity within the analyzed corpus, the 1436 documents were categorized based on VOSviewer metadata and keyword analysis. Approximately 65% (*n* = 934) of the documents are observational studies (e.g., cohort, cross-sectional, case-control), reflecting the feasibility of studying dietary patterns like breakfast consumption in large populations. Randomized controlled trials (RCTs) account for approximately 25% (*n* = 359), focusing on interventions such as breakfast composition or timing. The remaining 10% (*n* = 143) include meta-analyses, animal models, and other designs. Studies explicitly addressing underrepresented groups, such as ethnic minorities, low-income populations, children, or elderly individuals, are limited, with approximately 8% (*n* = 115) including such populations, often in the context of public health interventions or dietary surveys. For example, Basu et al. (2015) [40] examined dietary patterns in low-income communities, highlighting breakfast’s role in reducing cardiometabolic risk. The predominance of observational studies and limited focus on underrepresented groups suggests gaps in experimental evidence and diversity, which are further explored in the discussion of limitations.

## 4. Discussion

The bibliometric analysis of 1436 documents reveals significant trends in chrononutrition and cardiometabolic health, with the United States, Japan, and the United Kingdom leading in publication volume and impact. However, several limitations in the corpus warrant consideration. Approximately 30% of studies (*n* = 430), particularly RCTs, report small sample sizes (e.g., <100 participants), limiting statistical power and generalizability, as seen in studies like Farshchi et al. [23] and Nagao et al. [33]. Selection bias is noted in 25% of observational studies (*n* = 234), often due to reliance on self-reported dietary data, as exemplified by Mekary et al. [22] and Cahill et al. [32].

Additionally, only 8% of studies (*n* = 115) focus on underrepresented groups, such as ethnic minorities or low-income populations, with most studies targeting general adult populations, potentially limiting applicability to diverse groups (e.g., Basu et al. [40]).

The lack of diversity in study populations, combined with methodological constraints like selection bias and small sample sizes, underscores the need for larger, more inclusive RCTs and robust observational designs to enhance the generalizability of findings in breakfast-related cardiometabolic research. These limitations highlight opportunities for future research to address gaps in study design and population representation.

Leading journals like American Journal of Clinical Nutrition (53 documents, 6264 citations) and Nutrients (52 documents, 1267 citations) drive knowledge dissemination, as shown in Table 1, with studies like Mekary et al. [22] and Farshchi et al. [23] illustrating the impact of breakfast skipping and composition on type 2 diabetes and obesity risk. The United States, Japan, and the United Kingdom lead in publication volume (307, 166, and 117 documents, respectively), with high-impact studies like Aune et al. [31] and Cavalot et al. [50] emphasizing whole-grain consumption and postprandial glucose control. 

### 4.1. Clinical Relevance of Breakfast in Cardiovascular Health

This bibliometric analysis reveals that breakfast is not merely a dietary practice but a modulator of multiple physiological processes related to cardiovascular health. Breakfast omission is associated with a higher prevalence of risk factors such as obesity, insulin resistance, dyslipidemia, and elevated blood pressure (Mekary et al. [22]; Smith et al. [21]). These findings align with longitudinal studies suggesting a cumulative relationship between breakfast habits and metabolic health across the lifespan.

Conversely, breakfasts rich in dietary fiber, whole grains, fruits, β-glucans, and antioxidant compounds exert beneficial effects on lipid profiles, postprandial glycemia, and inflammation levels (Wolever et al. [20]; Ballesteros et al. [26]; AbuMweis et al. [35]). These physiological effects have been documented in both healthy populations and patients with type 2 diabetes and dyslipidemia, highlighting breakfast’s clinical applicability as a preventive tool.

Additionally, some studies point to breakfast’s specific role in modulating the cortisol-insulin axis and regulating appetite, which may explain its effects on body weight and energy metabolism (Missimer et al. [27]; Hollman et al. [17]). This hormonal dimension opens avenues for further research in nutritional endocrinology.

The focus on breakfast as a key factor in chrononutritional research is justified by its prominence in the VOSviewer analysis, with keywords like “meal” and “diet” appearing in 48% of documents (*n* = 689), reflecting its role in circadian alignment and metabolic regulation. Breakfast influences postprandial glucose and insulin sensitivity, as shown in studies like Cavalot et al. [50], which highlights its cardiometabolic impact. However, multiple factors contribute to these outcomes. Meal timing, including early time-restricted feeding (eTRF), affects circadian rhythms, with 15% of studies (*n* = 215) addressing “circadian rhythm” or “timing” (Jamshed et al. [25]). 

### 4.2. Historical Evolution and Field Maturation

The historical evolution shows that the literature on breakfast and cardiovascular health has progressed through three phases: a formative stage (1957–1985), a consolidation phase (1986–2004), and a period of scientific maturity (2005–2021). In the early decades, studies were clinical and limited in number, focusing on breakfast’s acute effects on lipids or glycemia (Wilkinson, [11]; Nicolaysen & Westinnd, [12]). Over time, methodological and population approaches diversified, with notable studies linking breakfast to psychosocial factors, eating behavior, and quality of life.

The 2005–2016 period saw the highest citation accumulation, indicating that articles from this phase served as conceptual foundations for subsequent research. This pattern suggests that the scientific community recognizes breakfast as a marker of healthy lifestyles, beyond a standalone dietary variable. The recent decline in publications (2024–2025) may be attributed to indexing delays rather than waning scientific interest.

### 4.3. Geographic Leadership and Collaborative Networks

Geographically, the United States leads in both publication volume and citations, followed by the United Kingdom, Japan, Canada, and Australia. This dominance aligns with their high investment in nutritional and epidemiological research (Aune et al. [31]; Phillips et al. [29]). The growth of contributions from the Global South, such as Mexico, Brazil, and, to a lesser extent, Colombia and Ecuador, is also noteworthy. While their participation remains nascent, the increase in publications reflects growing regional awareness of dietary patterns’ health impacts.

Co-authorship networks reveal that knowledge is largely generated through international collaborations. Prominent clusters include Anglo-Saxon (United States, United Kingdom, Canada), Asian (Japan, China, India), and European (Spain, Italy, Germany) groups, facilitating the exchange of clinical, social, and behavioral approaches. These collaborations enhance the field’s multidimensionality and make findings more applicable across diverse contexts. However, South-South collaboration networks, particularly in Latin America, Africa, and Southeast Asia, need further strengthening.

### 4.4. Emerging Themes and Conceptual Innovation

One of this study’s key contributions is identifying emerging thematic areas in both author and indexed keywords. Beyond the traditional focus on cholesterol, glucose, and blood pressure, there is growing interest in areas like chrononutrition, digital health, and prevention from early life stages (Jamshed et al. [25]; Joung et al. [46]; Cavalot et al. [50]).

Notably, early time-restricted feeding (eTRF) has gained prominence as a metabolic modulation strategy based on aligning breakfast with circadian rhythms. Recent studies show that early breakfast—and avoiding late-night eating—improves insulin sensitivity and reduces inflammatory biomarkers, even without altering total caloric intake (Jamshed et al. [25]).

Additionally, efforts to incorporate technologies such as mobile apps, glucose sensors, and continuous monitoring platforms in breakfast evaluation are evident. These advancements enable capturing individualized responses to different breakfast compositions and timings, supporting the shift toward personalized, data-driven nutrition (Zhang et al. [1]).

### 4.5. Gaps and Methodological Challenges

Despite progress, significant gaps persist. Most studies are observational and cross-sectional, limiting causal inference. The scarcity of well-designed randomized controlled trials hinders robust recommendations on the type, content, and timing of breakfast for specific populations.

Another limitation is the underrepresentation of vulnerable groups: few studies address breakfast in older adults, adolescents in school settings, or rural populations with food insecurity. The literature remains biased toward urban middle-class populations, potentially reducing the global applicability of recommendations.

Finally, heterogeneity in defining a “healthy breakfast” across studies complicates inter-study comparisons. Standardizing breakfast quality indicators, considering nutritional density, glycemic load, dietary diversity, and sociocultural factors, would be beneficial.

### 4.6. Cardiometabolic Risk Markers and Breakfast

To address the associations between breakfast consumption and cardiometabolic risk markers, this study identified several key markers frequently studied in the literature, including obesity, insulin resistance, dyslipidemia, blood pressure, and glycemic control. The analysis revealed that breakfast omission is consistently linked to adverse cardiometabolic outcomes. For instance, Smith et al. [21] found that skipping breakfast in childhood and adulthood is associated with greater waist circumference, elevated basal insulin, and higher total and LDL cholesterol levels, even after adjusting for diet and lifestyle. Similarly, Mekary et al. ([22]) reported a 21% higher risk of type 2 diabetes among breakfast skippers in a cohort of over 29,000 U.S. men, independent of body mass index and diet quality. Farshchi et al. [23] demonstrated that omitting breakfast for 14 days impairs insulin sensitivity and increases total and LDL cholesterol in healthy young women, providing experimental evidence of immediate physiological effects.

Conversely, breakfasts rich in fiber, whole grains, and antioxidants show protective effects. Wolever et al. (2010) found that daily intake of 3 g of high-molecular-weight oat β-glucans reduced LDL cholesterol by approximately 5% compared to wheat cereals, highlighting the role of breakfast composition in lipid management. Ballesteros et al. (2015) reported that daily egg consumption, compared to an oatmeal breakfast, reduced inflammatory markers like TNF-α and AST in type 2 diabetes patients without adversely affecting glucose or lipid profiles. Jamshed et al. [24] further emphasized the role of breakfast timing, showing that early time-restricted feeding (eTRF) improved 24-h glycemic control and increased expression of longevity-related genes, underscoring the chrononutritional benefits of early breakfast consumption.

These findings indicate that breakfast influences a range of cardiometabolic risk markers, with both observational and experimental studies supporting its role in metabolic health. However, the predominance of observational studies limits causal inferences, and further randomized controlled trials are needed to establish specific recommendations for breakfast composition and timing.

### 4.7. Public Health Implications and Recommendations

The literature highlights significant public health implications of breakfast consumption for cardiometabolic health, particularly in school, community, and clinical settings. Regular breakfast consumption is associated with lower obesity rates and healthier metabolic profiles, as emphasized by St-Onge et al. [19] in their American Heart Association statement. This suggests that promoting breakfast could be a key strategy in cardiovascular disease prevention. For example, Aune et al. [31] found that whole-grain consumption at breakfast reduces coronary heart disease risk by up to 20%, supporting the inclusion of whole grains in dietary guidelines.

While this study did not specifically analyze policies evaluating breakfast programs, the literature suggests actionable implications for public health interventions. School-based breakfast programs, as noted in studies addressing pediatric populations (e.g., Bano et al. [2]), can improve cognitive performance and cardiometabolic health by providing nutrient-dense breakfasts rich in fiber, fruits, and whole grains. Community-level interventions could focus on equitable access to healthy breakfast foods, particularly for rural or marginalized populations, as highlighted by the underrepresentation of these groups in the literature. Clinical settings could integrate chrononutrition principles, encouraging early breakfast consumption to align with circadian rhythms, as supported by Jamshed et al. ([25]).

To translate these findings into practice, public health strategies should prioritize nutritional education on breakfast’s role in metabolic health, develop region-specific dietary guidelines, and leverage technologies like mobile apps and continuous glucose monitoring for personalized interventions (Zhang et al. [1]). While direct evaluations of breakfast-specific policies were not a focus of this bibliometric analysis, the identified literature provides a foundation for designing evidence-based interventions to promote healthy breakfast habits across diverse populations.

## 5. Conclusions

This bibliometric analysis of 1436 documents (1957–2025), retrieved from Web of Science and Scopus after deduplication, underscores the dynamic role of breakfast in cardiometabolic health research. The United States, Japan, and the United Kingdom lead in publication volume and impact, contributing 307, 166, and 117 documents, respectively, with journals like American Journal of Clinical Nutrition and Nutrients driving dissemination. Key themes, including type 2 diabetes, obesity, glycemic control, and dietary flavonoids, highlight breakfast’s multifaceted impact, with 65% of studies (*n* = 934) being observational and 25% (*n* = 359) randomized controlled trials (RCTs). Dietary flavonoids, found in breakfast foods like berries and tea, modulate gut microbiota, enhancing metabolic outcomes, while cultural variations (e.g., fruit-based breakfasts in the UK vs. rice-based in Japan) and temporal shifts (1950s processed cereals vs. 2020s whole-grain options) influence health benefits. Only 8% of studies (*n* = 115) address underrepresented groups, revealing a critical research gap. These findings emphasize the need for public health strategies promoting balanced, flavonoid-rich breakfasts tailored to diverse populations and cultural contexts. Future research should prioritize larger, inclusive RCTs to strengthen experimental evidence, explore gut microbiota mechanisms, and address underrepresented groups like ethnic minorities and low-income populations. Additionally, investigating the interplay of meal timing, macronutrient balance, and regional dietary patterns will enhance the global applicability of chrononutritional interventions, advancing precision nutrition for cardiometabolic health.

## Figures and Tables

**Table 1:** Scientific journals with the highest production and impact in studies on breakfast and cardiovascular health. Lists the leading sources ordered by number of publications and citations received.

Source Title	Documents	Citations
American Journal of Clinical Nutrition	53	6264
Nutrients	52	1267
British Journal of Nutrition	31	1350
European Journal of Clinical Nutrition	26	1660
Diabetes Care	22	1703
Journal of Nutrition	22	1406
Nutrition Research	18	380
Journal of the American College of Nutrition	17	1294
PLoS ONE	16	541
Appetite	14	550

**Table 2:** Scientific production and collaboration networks by country in the field of breakfast and cardiovascular health. Details the number of documents, total citations, and total link strength based on bibliometric analysis using VOSviewer.

Country	Documents	Citations	Total Link Strength
United states	307	17,976	154
Spain	70	1958	80
Japan	166	3901	74
United Kingdom	117	5775	74
Germany	48	1821	73
Italy	57	2529	69
China	89	1161	63
Belgium	17	445	58
Sweden	45	2277	53
Australia	62	3071	48
Greece	22	512	47
Canada	58	3039	42
India	33	402	33
France	33	1678	32
Hungary	6	104	30
Poland	18	171	28
United Arab Emirates	7	72	28
Austria	8	277	27
Switzerland	21	1498	26

## Data Availability

The data supporting the findings of this study were retrieved from Scopus and Web of Science (WoS) databases using the following search equations: Scopus: TITLE-ABS-KEY (breakfast* AND “cardiovascular health” OR “cardiovascular disease” OR “heart disease” OR “cardiometabolic risk” OR “blood pressure” OR “cholesterol”), Web of Science (WoS): TS = (breakfast*) AND TS = (“cardiovascular health” OR “heart disease” OR “cholesterol” OR “blood pressure”). The selection of documents followed the inclusion and exclusion criteria detailed in the methodology section. While the raw bibliographic data (including metadata and abstracts) were obtained from licensed databases and are not publicly shareable due to copyright restrictions, the search strategies provided allow replication of the dataset retrieval process.
